# Research on Multi-Objective Optimization Method for Hydroforming Loading Path of Centralizer

**DOI:** 10.3390/ma18143310

**Published:** 2025-07-14

**Authors:** Zaixiang Zheng, Zhengjian Pan, Hui Tan, Feng Wang, Jing Xu, Yiyang Gu, Guoheng Li

**Affiliations:** School of Mechanical Engineering, Yangzhou University, Yangzhou 225000, China; tanhuiyzdx@163.com (H.T.); wangff0126@163.com (F.W.); jingxu@yzu.edu.cn (J.X.); 241202110@stu.yzu.edu.cn (Y.G.); 241405407@stu.yzu.edu.cn (G.L.)

**Keywords:** tube hydroforming, centralizer, loading path, multi-objective optimization

## Abstract

During centralizer hydroforming, internal pressure and axial feed critically influence the forming outcome. Insufficient feed causes excessive thinning and cracking, while excessive feed causes thickening and wrinkling. Achieving uniform wall thickness necessitates careful design of the pressure and feed curves. Using max/min wall thickness as objectives and key control points on these curves as variables, the study integrated Non-dominated Sorting Genetic Algorithm (NSGA-II), Multi-Objective Particle Swarm Optimization (MOPSO), Neighborhood Cultivation Genetic Algorithm (NCGA), and Archive-based Micro Genetic Algorithm (AMGA) with LS-DYNA to automatically optimize loading paths. The results demonstrate the following: ① NSGA-II, NCGA, and AMGA successfully generated optimized paths; ② NSGA-II and AMGA produced larger sets of higher-quality Pareto solutions; ③ AMGA required more iterations for satisfactory Pareto sets; ④ MOPSO exhibited a tendency towards premature convergence, yielding inferior results; ⑤ Multi-objective optimization efficiently generated diverse Pareto solutions, expanding the design space for process design.

## 1. Introduction

The centralizer plays a pivotal role in stabilizing drilling direction for oil, gas, and geological exploration drilling projects. Its central cross-sectional circumference is 20% larger than the cross-sectional circumference of the ends, and in extreme cases, it is 40% larger. During hydroforming, precise matching between the fluid pressure inside the pipe blank and axial feed displacement at both ends is critical; improper matching can easily cause forming failure, necessitating meticulous design. However, aligning these parameters through theoretical analysis, empirical data, trial-and-error testing, and numerical simulation requires substantial testing—a time-consuming and labor-intensive process. Moreover, it is challenging to attain the optimal matching relationship within a limited timeframe. Consequently, novel matching optimization methodologies must be developed. Marlapalle, B. G. et al. [1] employed numerical simulation and experimental methods to investigate the hydroforming process of pipe fittings and to predict the formability parameters of stainless steel AISI304 and AISI409L. Zhang, C. et al. [2] employed response surface methodology to analyze the effects of various loading path factors on the forming performance of double-convex tubes and proposed linear loading as the optimal loading method. Raut, S. V. et al. [3] defined elastic-plastic material behavior through flow stress equations and applied the Arbitrary Lagrange–Eulerian (ALE) technique to characterize material flow. They also optimize the hydroforming process using the Design of Experiment (DOE) technique–response surface methodology. Jiang, X. et al. [4] used a Kriging model to approximate and quantify the relationship between variable press force and forming defects in the sheet metal forming process, and optimized the process parameters using an improved QO-Jaya algorithm. Ghorbani-Menghari, H. et al. [5] used experimental and numerical simulation methods to optimize hydroforming process parameters with the help of a single-objective genetic algorithm. El-Aty, A. A. et al. [6] proposed a method for multi-step hydroforming of thin-walled five-branch stainless steel pipes based on the response surface method, which was verified through simulation analysis and testing. Trung, N. D. et al. [7] conducted numerical simulations based on ABAQUS/CAE software to study the effects of three prominent inclination angles (45°, 60°, and 90°) on the formability of hollow joints HJ45, HJ60, and HJ90. Zheng, Z. [8] was the first to apply the NSGA-II multi-objective optimization algorithm to the optimization of hydroforming process parameters. However, due to the limitations of the computational hardware and software at the time, large-scale iterative computations were not feasible. As a result, the obtained Pareto-optimal solutions were limited in both quality and quantity, indicating the need for further improvement. Subsequently, Zheng, Z. et al. [9] integrated NSGA-II with LS-DYNA to optimize hydroforming process parameters and systematically investigated the influence of parameters, such as population size, number of generations, and iteration count, on the distribution of Pareto-optimal solutions. Considering factors such as time efficiency and cost effectiveness, they identified the optimal number of iterations and the corresponding Pareto-optimal solution set for a specific instrument panel tube beam forming case. Ghulman, H. A. et al. [10] applied particle swarm optimization to generate hydroforming parameter datasets, enhancing Pareto front solution accuracy through extended convergence time. Abdessalem, A. B. et al. [11] developed a probabilistic method for optimizing hydroforming structural variability, achieving uniform wall thickness distribution in T-shaped pipes via simulations and experiments. Based on the optimal process parameters for hydroforming 316L stainless steel T-joints, Fiorentino, A. et al. [12] established a comprehensive objective function for billet length and thickness, used linear search and binary search methods to optimize the function, and determined the optimal dimensions for the billet. Ge, Y. L. et al. [13] proposed an adaptive method based on fuzzy logic theory to plan the loading path for hydroforming asymmetrical pipe fittings, which was successfully applied to the hydroforming of asymmetrical pipe fittings, etc.

In addition to its extensive application in tube hydroforming, multi-objective optimization has also been widely employed across various sectors of the manufacturing industry. Marcineková, K. et al. [14] applied Artificial Neural Networks (ANN) in the furniture manufacturing process, optimizing key production steps based on three sets of input and output parameters. Their objective was to promote sustainable process improvement by minimizing resource consumption and waste, while enhancing overall system efficiency. Park, J. et al. [15] conducted a multi-objective optimization of facility layout planning within automotive cellular manufacturing systems. They implemented and compared simulated annealing, particle swarm optimization, and NSGA-II, aiming to improve both productivity and flexibility in automotive manufacturing environments. Pak, N. et al. [16] developed a multi-product, five-echelon green supply chain network, prioritizing the minimization of transportation and construction costs, followed by the reduction of CO_2_ emissions across various supply chain levels. They employed and compared NSGA-II and SPEA-II, concluding that SPEA-II outperformed NSGA-II across all evaluation metrics. Hsieh, T. J. [17] proposed an indicator-based ε-dominance Multi-Objective Artificial Bee Colony (ε-MOABC), incorporating a novel encoding scheme and evolutionary operators. This approach effectively integrated production routing and machine selection to meet customized production requirements and was successfully validated through case studies, offering practical insights for resource allocation in diverse manufacturing settings. Ghodratnama, A. et al. [18] established a multi-objective optimization model with three objective functions for flow line production management in flexible manufacturing systems. The model was validated using both NSGA-II and MOPSO.

Current hydroforming loading path optimization primarily focuses on single objectives, conventional algorithms, limited design variables, and proxy models. These approaches suffer from local optima, low efficiency, and significant discrepancies between proxy models and actual conditions. This study addresses these limitations by establishing tube blank maximum and minimum wall thickness as optimization objectives. Key control points on the internal pressure and axial feed rate curves are selected as design variables. Four multi-objective optimization algorithms—NSGA-II, MOPSO, NCGA, and AMGA—are integrated with LS-DYNA. The direction of the curves is altered by modifying the values of the key control points on the internal pressure loading curve and axial feed rate curve via computer simulation. This process enables optimization within an expanded solution space. Simulation results from these four multi-objective design methods are comparatively analyzed to identify effective approaches for centralizer loading path design and Pareto optimal solution sets.

## 2. Structural Features

**Figure 1:**
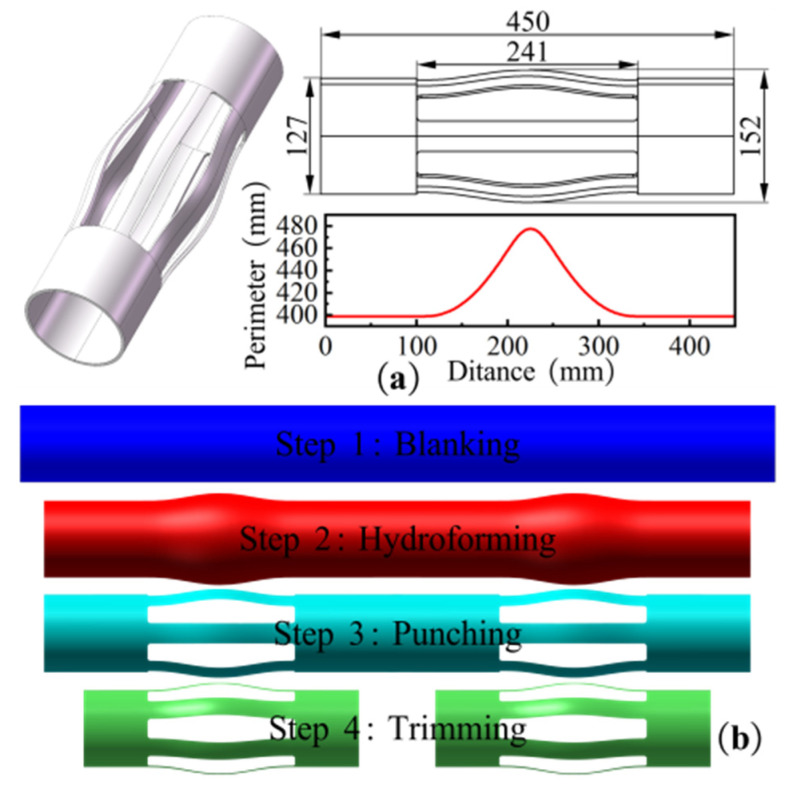
(**a**) Cross-section perimeter of centralizer; (**b**) Forming process of centralizer.

The integral elastic centralizer has an axial length of 450 mm, with an elastic protrusion length of 241 mm and a wall thickness of 4 mm. The circumference and shape characteristics along the axial cross section are illustrated in Figure 1a. The maximum circumference of the cross-section is approximately 477.5 mm (diameter *ϕ*152 mm), and the minimum diameter at both ends is 399 mm (diameter *ϕ*127 mm), representing a discrepancy of 19.7%. Given the symmetrical nature of the centralizer and its simple geometric form, a strategy was devised to process two pieces concurrently. This approach has been shown to enhance equipment productivity by more than 90% compared to single-piece processing methodologies. As illustrated in Figure 1b, the hydroforming process of the centralizer is as follows:

1. Cutting: Cut high-strength seamless steel pipes to the designed length.
2. Hydroforming: The expansion process is then initiated by placing the tube blank within a hydroforming machine.
3. Punching: Laser cutting is used to cut six holes.
4. Trimming: Use wire cutting to cut the ends of the centralizer.

## 3. Models, Methods, and Experiments

### 3.1. Finite Element Modeling and Methods

#### 3.1.1. Numerical Simulation Analysis Model of Hydroforming

**Figure 2:**
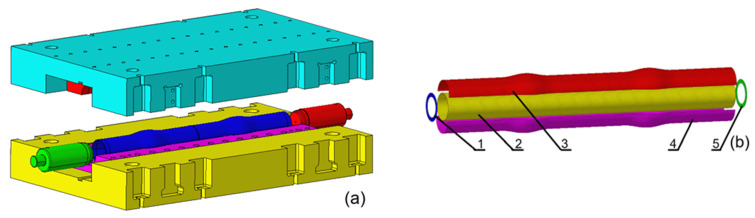
Finite element model of centralizer: (**a**) Geometric model; (**b**) Finite element model: 1. Left plug; 2. Tube blank; 3. Upper die; 4. Lower die; 5. Right plug.

The external diameter of the tube blank is measured at 127 mm, with a wall thickness of 4.0 mm. Simultaneous processing of two pieces is a key aspect of the manufacturing process. In consideration of the pertinent factors, including product specifications, length, axial feed rate, sealing and transition sections, cutting volume, and raw materials, the preliminary length of the tube blank is established at 1450 mm. During the simulation, the surfaces of the die cavity and the end plugs of the tube blank are employed to represent the die and plugs, and they are set as rigid bodies. Considering the computational efficiency and the accuracy of the model, 6 mm elements are adopted. The die and plugs are divided using in-plane single-point integral Belytschko–Tsay elements. The tube blank is a flexible body that uses in-plane full-integration Belytschko–Tsay elements for mesh generation. The total number of elements is 30,143, among which the number of elements for the tube blanks is 14,175. The contact between the tube blanks, the dies, and the plugs is achieved through one-way face-to-face contact, as well as the self-contact of the tube blanks on one side. The material is 40CrMo, described using a multi-segment linear elastic-plastic isotropic strengthening model. The constitutive equation satisfies the relationship $\bar{\sigma }=1470{\left(0.0002+\bar{\varepsilon }\right)}^{0.214}$ is employed to denote the relationship between equivalent stress $\bar{\sigma }$ and equivalent strain $\bar{\varepsilon }$. The established geometric model and finite element model are illustrated in Figure 2.

#### 3.1.2. The Selection of Numerical Simulation Algorithms

In comparison with static implicit algorithms, dynamic explicit algorithms utilize explicit time integration schemes, which simulate the motion and deformation of objects through incremental solution of the dynamic equations of the system. The formation of stiffness matrices is not a prerequisite for this method, which has been demonstrated to efficiently address complex nonlinear problems, including contact, plastic deformation, large translations, and large rotations. This capacity renders the method well-suited for simulating the hydroforming process of centralizers. The governing dynamical equations, derived from D’Alembert’s principle of virtual work are expressed as follows: [19].

(1) $$M\ddot{u}+C\dot{u}+Ku=F_{\mathrm{ext}}\left(t\right)$$

where $F_{\mathrm{ext}}\left(t\right)$ is the external load; M, C, and K are the mass, damping, and stiffness matrices, respectively; u, $\dot{u}$, and $\ddot{u}$ are the nodal displacement, velocity, and acceleration vectors, respectively.

This equation can be solved using the dynamic explicit center difference method as follows:

(2) $${\dot{u}}_{t}=\frac{1}{2\Delta t}\left(u_{t+\Delta t}-u_{t-\Delta t}\right)$$

(3) $$\begin{array}{cc} {\ddot{u}}_{t} & =\frac{1}{\Delta t}\left({\dot{u}}_{t+\frac{\Delta t}{2}}-{\dot{u}}_{t-\frac{\Delta t}{2}}\right)\\ & =\frac{1}{\Delta t}\left(\frac{u_{t+\Delta t}-u_{t}}{\Delta t}-\frac{u_{t}-u_{t-\Delta t}}{\Delta t}\right)\\ & =\frac{1}{\Delta t^{2}}\left(u_{t+\Delta t}-2u_{t}+u_{t-\Delta t}\right) \end{array}$$

Substituting Equations (2) and (3) into Equation (1) gives the following:

(4) $$\left(M+\frac{1}{2}\Delta tC\right)u_{t+\Delta t}=\Delta t^{2}F_{ext}\left(t\right)-\left(\Delta t^{2}K-2M\right)u_{t}-\left(M-\frac{1}{2}\Delta tC\right)u_{t-\Delta t}$$

(5) $$u_{t+\Delta t}=\frac{F_{ext}\left(t\right)-\left(K-\frac{2}{\Delta t^{2}}M\right)u_{t}-\left(\frac{1}{\Delta t^{2}}M-\frac{1}{2\Delta t}C\right)u_{t-\Delta t}}{\frac{1}{\Delta t^{2}}M+\frac{1}{2\Delta t}C}$$

Based on the nodal displacements, the nodal velocity gradient tensor $\nabla \dot{u}$ and strain can be solved as follows:

(6) $$\nabla \dot{u}=\left[\begin{array}{ccc} \frac{𝜕{\dot{u}}_{x}}{𝜕x} & \frac{𝜕{\dot{u}}_{x}}{𝜕y} & \frac{𝜕{\dot{u}}_{x}}{𝜕z}\\ \frac{𝜕{\dot{u}}_{y}}{𝜕x} & \frac{𝜕{\dot{u}}_{y}}{𝜕y} & \frac{𝜕{\dot{u}}_{y}}{𝜕z}\\ \frac{𝜕{\dot{u}}_{z}}{𝜕x} & \frac{𝜕{\dot{u}}_{z}}{𝜕y} & \frac{𝜕{\dot{u}}_{z}}{𝜕z} \end{array}\right]$$

(7) $$\dot{\varepsilon }=\frac{1}{2}\left[\nabla \dot{u}+{\left(\nabla \dot{u}\right)}^{T}\right]$$

(8) $$\Delta \varepsilon =\Delta t\cdot \dot{\varepsilon }$$

where $\dot{\varepsilon }$ is the material integral strain rate; Δε is the element strain increment.

The stress at the next moment can be found by substituting Δε into the intrinsic equation of the material:

(9) $$\sigma_{t+\Delta t}=f\left(\sigma_{t},\Delta \varepsilon \right)$$

The aforementioned solution process does not necessitate matrix inversion, and the stability and accuracy of the calculation can be assured if the time step satisfies the critical condition. Consequently, in the hydroforming process, the dynamic explicit center difference method is employed to solve the kinetic equations, thereby ensuring the attainment of optimal forming results.

#### 3.1.3. Load Path Design

**Figure 3:**
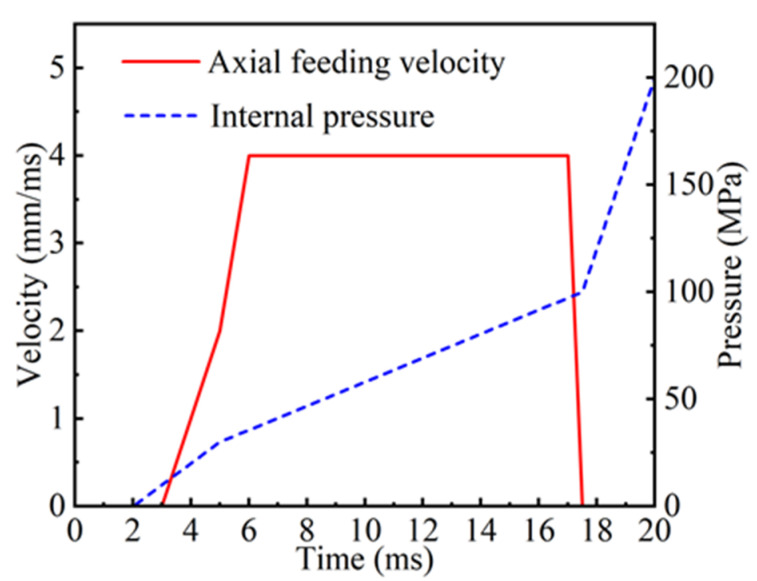
Axial feed rate and internal pressure loading curve.

The internal pressure curve comprises four segments, which represent the initial yield, forming, shaping, and pressure maintenance stages, respectively [20]. Of these, the forming stage and the shaping stage exert the most significant influence on process outcomes. In the former, the pressure increases gradually to facilitate the smooth formation of pipe fittings. In the latter, the pressure increases rapidly to reduce the forming time while ensuring that the shape of the parts is adjusted, thereby improving production efficiency. During the forming stage, rapid material replenishment is required to meet the needs of tube blank expansion. During the shaping stage, the degree of deformation that continues to occur in the tube blank is minimal, and the necessity for material replenishment is essentially negligible. In consideration of the factors enumerated below, including but not limited to equipment processing capacity, material mechanical properties, the interrelationships between variables, processing experience, and requirements, the design of internal pressure loading curves and axial feed speed curves was executed, as illustrated in Figure 3.

### 3.2. Optimization Models and Methods

#### 3.2.1. Determination of Design Variables

While both ends of the centralizer have a diameter of 127 mm, its central section measures 152 mm in diameter. During hydroforming, synchronized application of axial force at the pipe extremities with internal pressure buildup is critical. This ensures that the billet does not thin excessively or crack. Furthermore, this ensures that the replenishment head of the shaft end and the end of the pipe billet are in close contact with the inner wall and form a closed cavity. Consequently, it is imperative to meticulously design the internal pressure loading curve and axial feed curve. The crux of the issue pertains to the methodology for enabling the computer to autonomously adjust the pressure and feed curves during the integration and optimization processes. The proposed methodology entails the selection of specific control points along the curve, the subsequent designation of these points as design variables, and the realization of the change of pressure and feed curves through the manipulation of the coordinates of the aforementioned control points. This methodology is expressed as follows:

(10) $$P(t)=\left[P_{1},P_{2},\cdots ,P_{m}\left|t_{1},t_{2},\cdots ,\right.t_{m}\right]$$

(11) $$V(t)=\left[V_{1},V_{2},\cdots ,V_{n}\left|t_{1},t_{2},\cdots ,t_{n}\right.\right]$$

In the formula, $P_{1},P_{2},\cdots ,P_{m}$ is the control point on the internal pressure loading curve; $V_{1},V_{2},\cdots ,V_{n}$ is the control point on the axial feed speed curve; $t_{m}$ and $t_{n}$ are the time and quantity corresponding to the internal pressure and speed control points, respectively.

**Figure 4:**
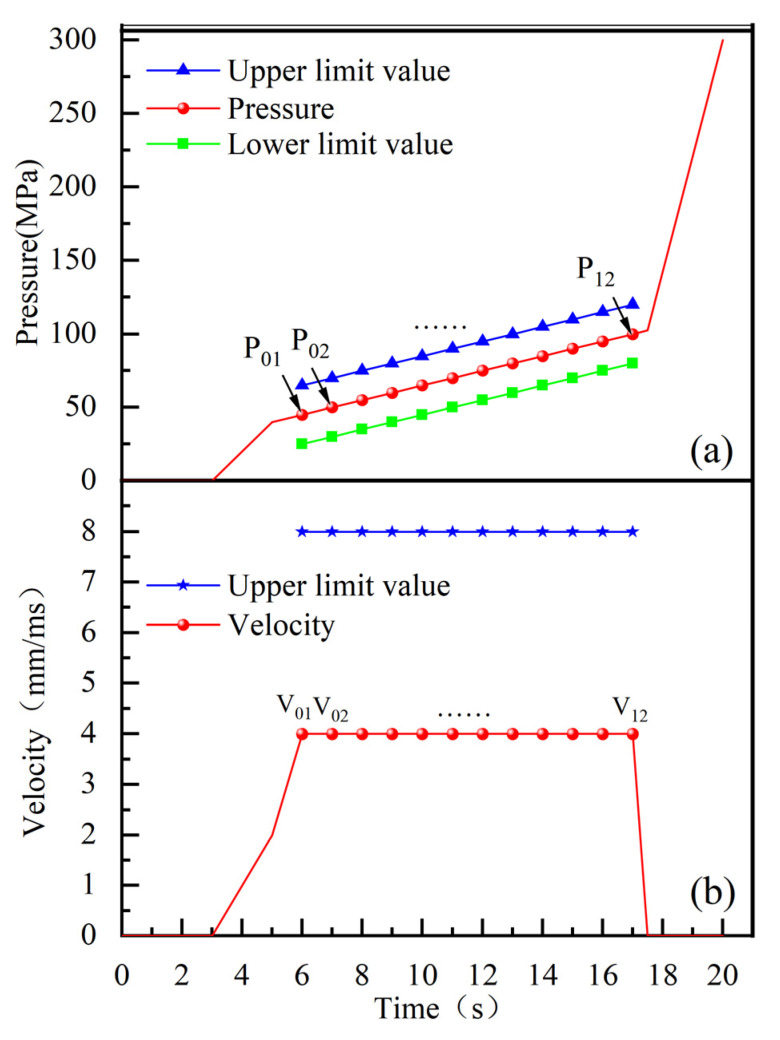
(**a**) Internal pressure control points and their boundaries; (**b**) Feed velocity control points and their boundaries.

The selection of 12 control points on both the pressure and speed curves was determined by the structural characteristics of the parts and the requirements of the forming process. This selection resulted in a total of 24 control points as design variables. The specific curves are illustrated in Figure 4.

#### 3.2.2. Determination of Objective Function

During the hydroforming process of the centralizer, local thickening or thinning is to be expected; however, excessive thickening or thinning should be avoided, as it can easily lead to local wrinkling or cracking. Consequently, it is imperative to exercise stringent control over the maximum and minimum wall thickness of the tube blank. The maximum and minimum thicknesses of the tube blank are designated as the target functions, which are expressed as follows:

(12) $$f_{1}\left(P\left(t\right),V\left(t\right)\right)=\min\left(T_{\max}\right)$$

(13) $$f_{2}\left(P\left(t\right),V\left(t\right)\right)=\max\left(T_{\min}\right)$$

In the formula, $T_{\min}={\left\{T_{1},T_{2},\cdots ,T_{N}\right\}}_{\min}$; $T_{\max}={\left\{T_{1},T_{2},\cdots ,T_{N}\right\}}_{\max}$; $T_{i}$ is the thickness of the i element after forming; N is the total number of elements.

#### 3.2.3. Multi-Objective Optimization Method

**Figure 5:**
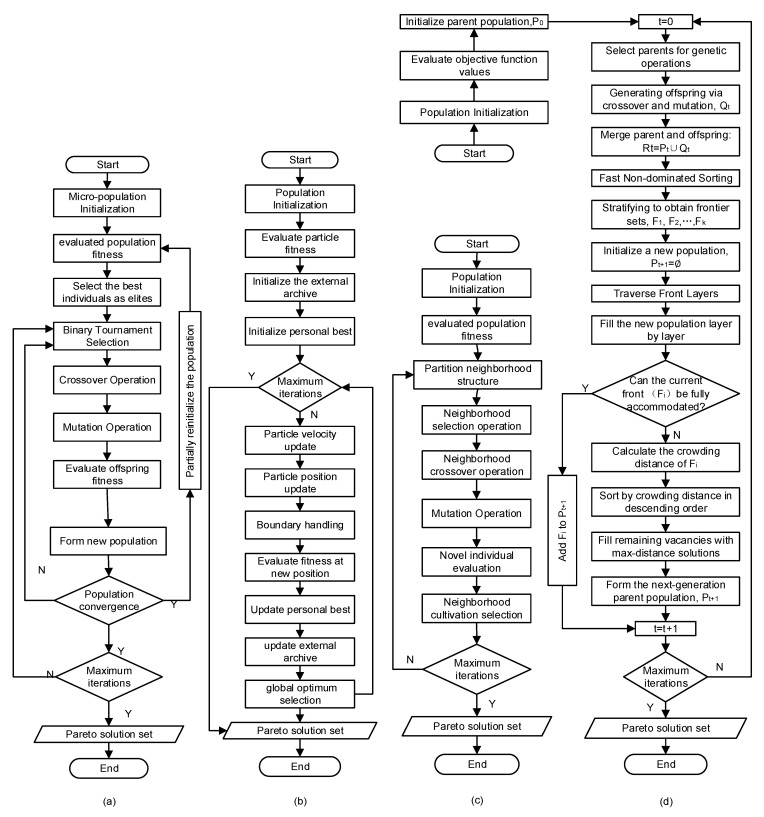
Four types of genetic algorithm flowcharts: (**a**) AMGA; (**b**) MOPSO; (**c**) NCGA; (**d**) (NSGA-II).

Common multi-objective optimization algorithms include AMGA, NCGA, MOPSO, and NSGA-II, among others. Among these, AMGA incorporates a dynamic crossover and mutation probability adjustment strategy, enabling adaptive control of search intensity based on the population state. NCGA maintains population diversity through niche engineering to prevent premature convergence. MOPSO employs a dynamic search strategy based on particle swarm collaboration, incorporating non-dominated sorting and inertia weight adjustment, balancing global and local search capabilities. NSGA-II employs fast non-dominated sorting and crowding degree calculation, achieving an elite retention strategy by merging parent and offspring populations, thereby ensuring solution diversity and convergence. All four algorithms demonstrate the capacity to address complex nonlinear problems. The integration of these four algorithms with LS-DYNA is intended to optimize the loading path of the centralizer hydroforming process. This integration is also intended to identify an optimization method that is more suitable for the hydroforming loading path. The flowcharts of AMGA, NCGA, MOPSO, and NSGA-II [21,22,23,24] are displayed in Figure 5.

#### 3.2.4. Multi-Objective Optimization Process

**Figure 6:**
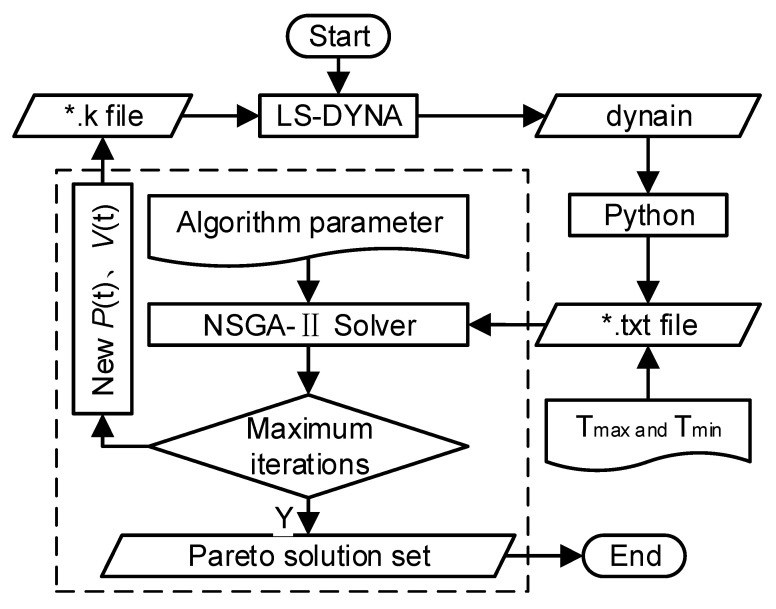
Multiple-objective optimization flowchart.

The four genetic algorithms operate under an identical workflow. Taking NSGA-II as an example, the specific steps are as follows: First, convert the 3D solid model into a finite element model and output it as a k file (containing design variables, control parameters, material models, etc.) and a mod file (containing node and element information, etc.). LS-DYNA reads the k file and mod file, performs hydroforming simulation analysis, and outputs a dynain file. Python 3.12.3 extracts and compares the shell element thicknesses in the dynain file, outputs the maximum and minimum thicknesses of the elements in txt format, the NSGA-II solver reads the maximum and minimum thicknesses of the elements, saves them, completes one iteration calculation, simultaneously updates the design variables in the k file, performs the next iteration calculation, and stops after reaching the maximum number of iterations. Finally, the NSGA-II solver searches for the optimal solution from all iteration results and outputs the Pareto optimal solution set. The multi-objective integrated optimization flowchart of the centralizer is shown in Figure 6.

#### 3.2.5. Experimental Design for Optimization

As the number of simulation iterations increases, the number of Pareto optimal solutions obtained also increases, and the solutions become progressively more refined. Conversely, this approach has been shown to result in longer computation times, lower time-efficiency ratios, and reduced cost-effectiveness. Therefore, it is necessary to determine the optimal number of iterations. The determination of the number of iterations is influenced by the implementation of various algorithms. For NCGA, MOPSO, and NSGA-II, the number of iterations is dependent on both the population size and the number of evolutionary generations, and is the product of these two values. However, for AMGA, the number of iterations is unrelated to the population size and the number of evolutionary generations. Instead, it is determined by the number of function evaluations. The configuration of the system is determined by the requirements of the analysis. To assess the impact of the four algorithms and iteration counts on optimization outcomes, this simulation experiment was designed with 16 schemes across four iteration levels, as illustrated in Table 1.

### 3.3. Actual Manufacturing Test

**Figure 7:**
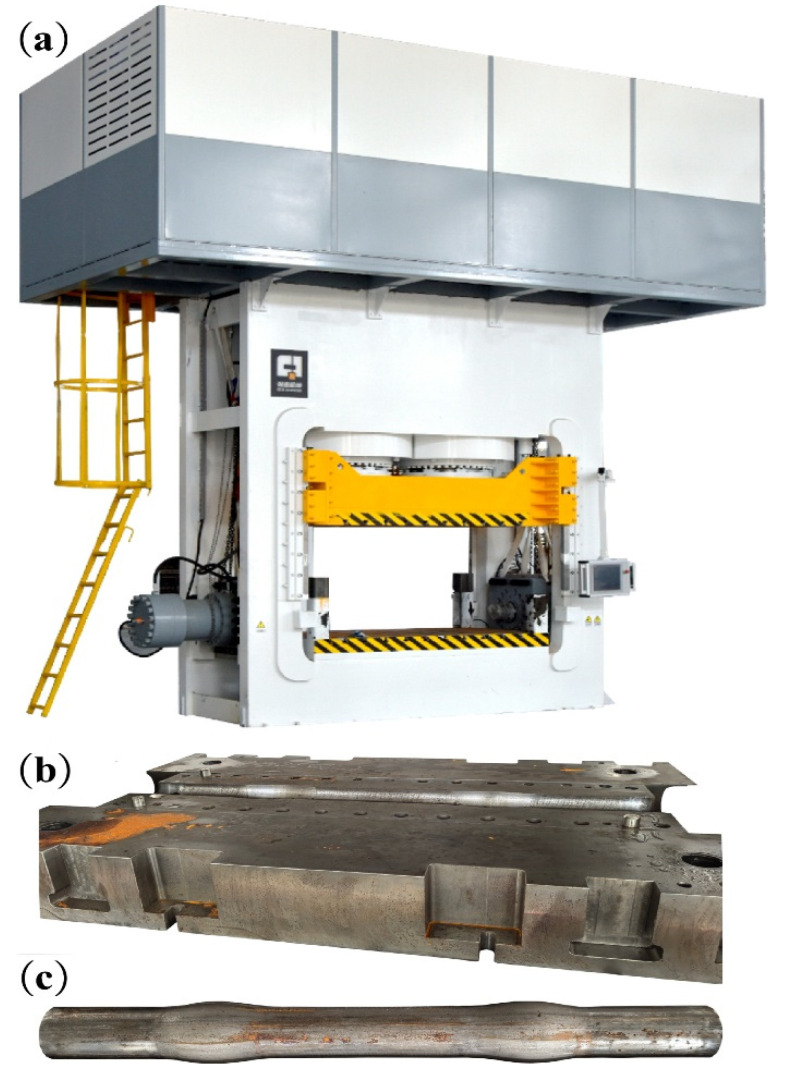
(**a**) Actual form of self-developed 2000T internal high-pressure forming machine; (**b**) Hydroforming lower die for centralizer actual form; (**c**) Tube blank after hydraulic expansion actual form.

The actual manufacturing test was conducted on a self-developed 2000 T hydroforming machine. The nominal clamping force of this equipment is 20,000 kN, the maximum forming pressure is 400 MPa, and the axial feed force and feed displacement are 2000 kN and 300 mm, respectively. Figure 7a–c show the centralizer die and the centralizer sample after hydroforming, respectively.

## 4. Results and Discussion

### 4.1. AMGA

**Figure 8:**
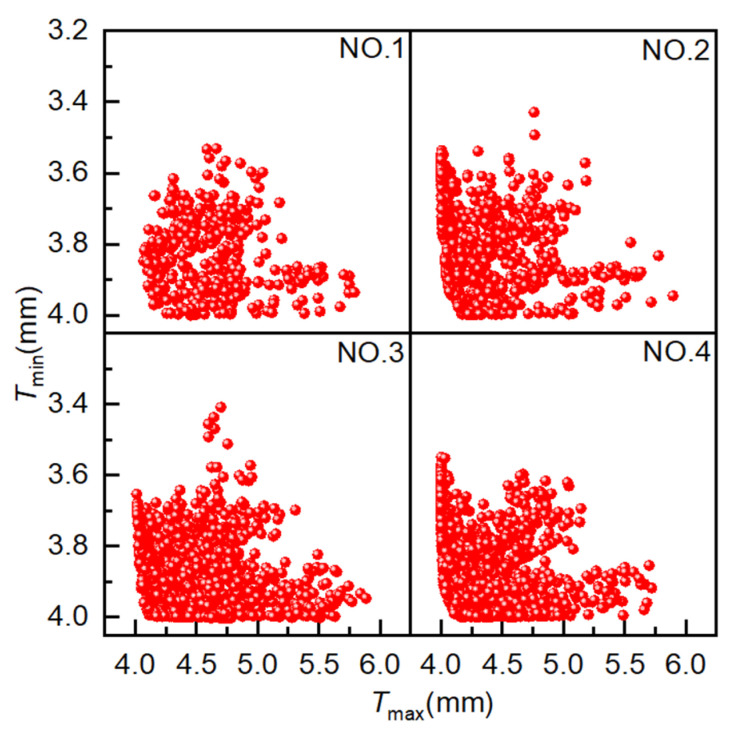
Scatter plot distribution of solutions obtained using AMGA.

The initial population size and evolutionary population size are set to 40, the archive size and Pareto front size are set to 100, the crossover probability is 0.9, the mutation probability is 0.5, and the evolutionary generation settings are as shown in Table 1. Figure 8 illustrates the simulation results from four experiments conducted using AMGA. The horizontal and vertical axes represent the maximum and minimum element thickness values, respectively, with each point representing a solution. The proximity of a point to the vertical axis is indicative of the following: a smaller pipe thickening rate, a lower wrinkling probability, a lighter weight, and a more pronounced weight reduction effect. Proximity to the horizontal axis corresponds to a smaller pipe thinning rate and superior forming quality. Consequently, when a point is proximate to both the horizontal and vertical axes (i.e., the origin), the obtained solution set approaches the optimal solution. As demonstrated in the accompanying figure, as the number of iterations increases, a growing number of solutions coalesce on the Pareto front, manifesting a discernible convergence trend. Concurrently, the optimal solution distribution range undergoes expansion, reflecting enhanced optimization performance.

**Figure 9:**
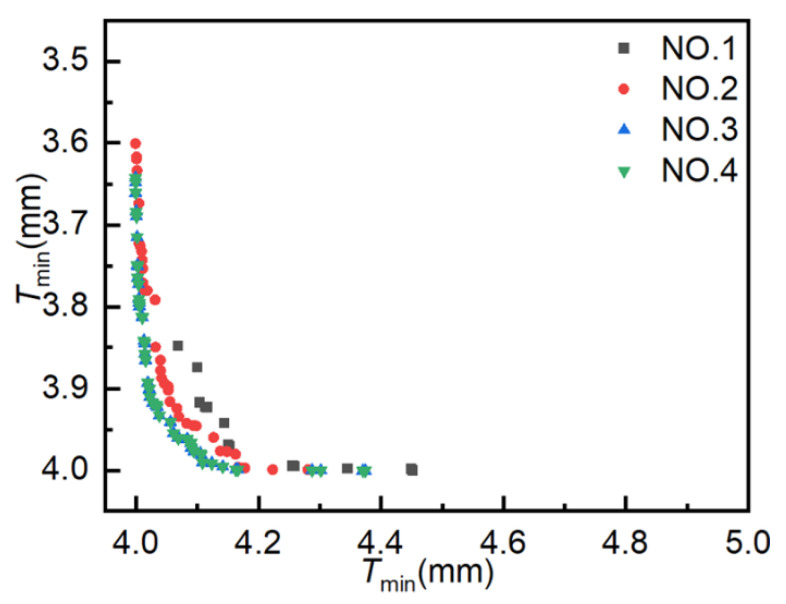
Pareto optimal solutions obtained from AMGA experiments.

Figure 9 illustrates the distribution of Pareto optimal solutions across the four sets of experiments. The following observations can be made from the figure: ① Results for 400, 800, 1200, and 1600 iterations yielded 14, 41, 61, and 61 Pareto optimal solutions, respectively. The number of Pareto optimal solutions increases with the number of iterations, but the increase is not significant in the later stages. ② As the number of iterations increases, the Pareto optimal solutions become increasingly optimal, with the Pareto optimal solutions obtained from Experiment NO. 4 being more optimal than those obtained from Experiment NO. 1. ③ When the number of iterations increases from 1200 to 1600, the Pareto frontiers of the two experiments are almost identical, with the same number of optimal solutions, indicating minimal room for further improvement. These results suggest that AMGA is effective in optimizing hydroforming process parameters, and that 1200 iterations represents an optimal balance between computational effort and optimization performance.

### 4.2. NCGA

**Figure 10:**
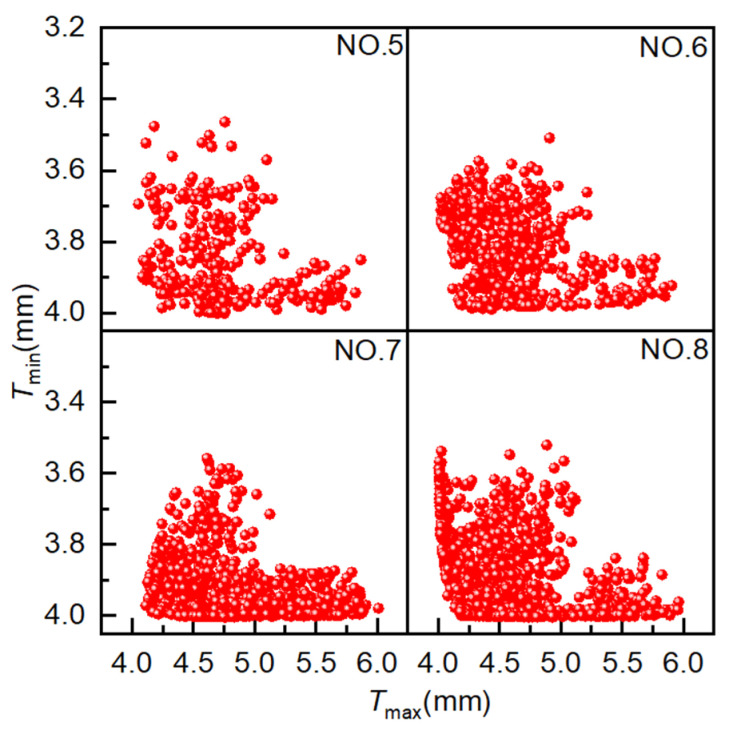
Scatter plot distribution of solutions obtained using NCGA.

The population size is 40, the crossover type is single-point crossover, the gene size is 20, the crossover probability is 1.0, the mutation probability is 0.01, and the evolutionary generation settings are as shown in Table 1. As illustrated in Figure 10, the simulation results of four simulation experiments employing NCGA are represented by points, with each point corresponding to a distinct solution. The coordinate settings are consistent with those employed for AMGA. As demonstrated in Figure 10, as the number of iterations increases, a greater number of solutions tend to congregate on the Pareto front, demonstrating marked convergence behavior. The optimal solutions are distributed over a broader range, indicating that the optimization performance is satisfactory.

**Figure 11:**
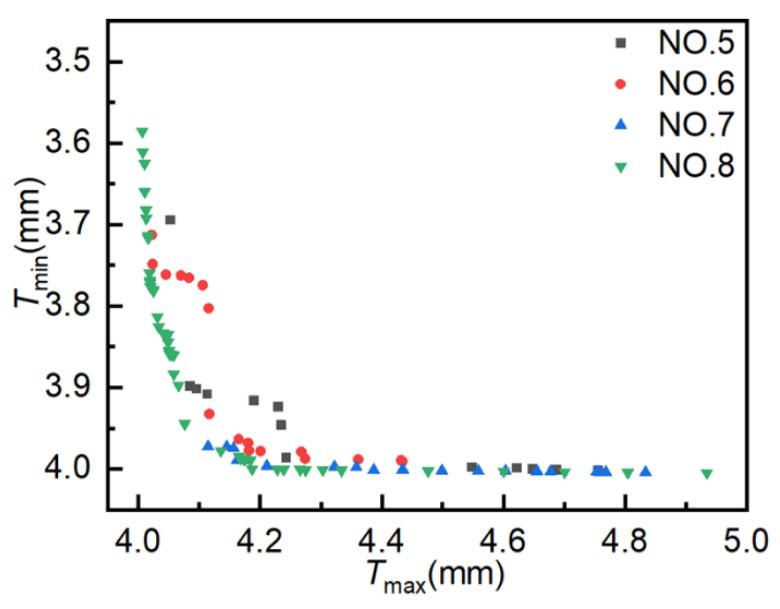
Pareto optimal solution under different experiments. Pareto optimal solutions obtained from NCGA experiments.

Figure 11 presents the distribution of Pareto optimal solutions for four sets of experiments. The following observations can be seen from the figure: ① Results for 400, 800, 1200, and 1600 iterations yielded 15, 17, 20, and 51 Pareto optimal solutions, respectively, with the number of Pareto optimal solutions increasing with the number of iterations. ② As the number of iterations increases, the Pareto optimal solutions become increasingly optimal, with those obtained from Experiment NO. 8 being more optimal than those from Experiment NO. 5. ③ As the number of iterations increases, the distribution range of optimal solutions becomes broader and more uniform. After the number of iterations increases from 1200 to 1600, the optimal solutions from both experiments converge on the Pareto front; however, the number of optimal solutions differs greatly. These results indicate that NCGA is capable of effectively optimizing hydroforming process parameters, with 1600 iterations identified as the most effective configuration in this study.

### 4.3. MOPSO

**Figure 12:**
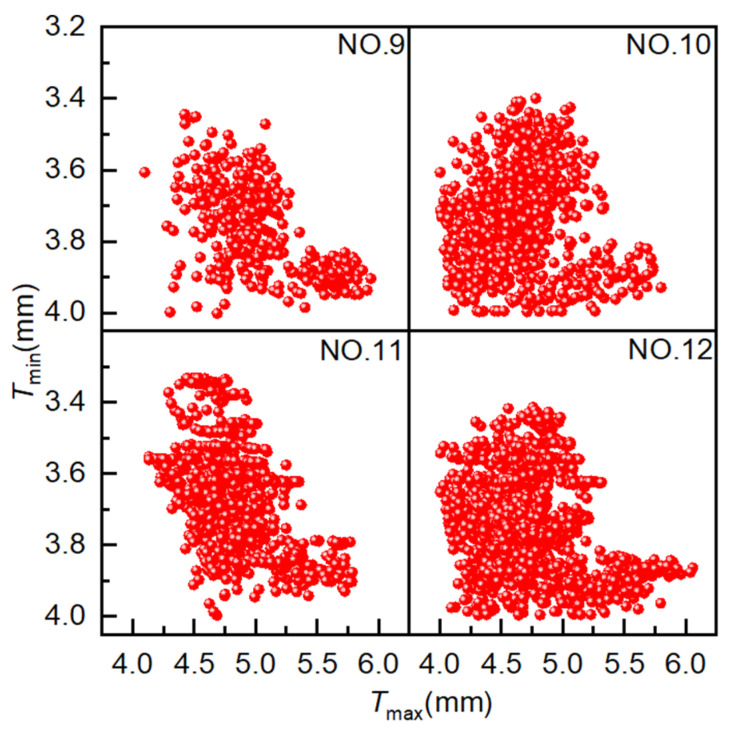
Scatter plot distribution of solutions obtained using MOPSO.

The population size is set to 10, the crossover type is single-point crossover, the gene size is 20, the crossover probability is 1.0, the mutation probability is 0.01, and the evolutionary generation settings are as shown in Table 1. As illustrated in Figure 12, the simulation outcomes of four distinct simulation experiments employing MOPSO are presented, with each data point representing a distinct solution. The coordinates for these solutions have been set to align with those utilized in the AMGA simulation. As shown in the figure, the distribution range of optimal solutions broadens with an increasing number of iterations. However, the optimization results are not optimal. The solutions from Experiment NO. 10 exhibited a greater concentration on the Pareto front compared to those from Experiment NO. 12.

**Figure 13:**
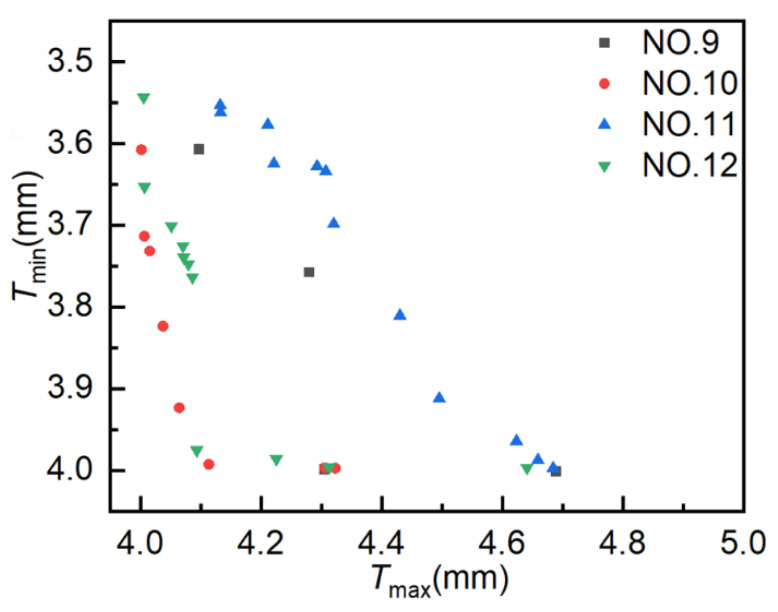
Pareto optimal solution under different experiments. Pareto optimal solutions obtained from MOPSO experiments.

Figure 13 presents the distribution of Pareto optimal solutions across four experimental sets. The following observations can be seen from the figure: ① Results for 400, 800, 1200, and 1600 iterations yielded 4, 8, 12, and 11 Pareto optimal solutions, respectively. The number of Pareto optimal solutions increases with the number of iterations, but not significantly in the later stages, and may even decrease. ② As the number of iterations increases, the Pareto optimal solutions do not tend to become more optimal. The Pareto optimal solutions obtained in experiment NO. 12 are of poorer quality than those obtained in experiment NO. 10. ③ The Pareto optimal solutions obtained in the four experiments are unevenly distributed and exhibit a tendency to converge toward local optima. These results suggest that MOPSO is not well-suited for optimizing hydroforming process parameters, due to its limited convergence accuracy and tendency to fall into local optima.

### 4.4. NSGA-II

**Figure 14:**
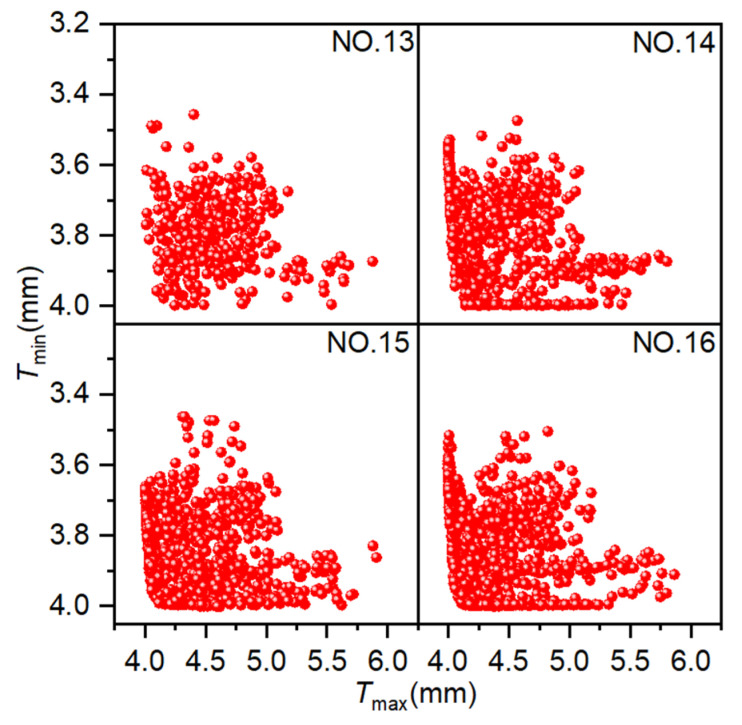
Scatter plot distribution of solutions obtained using NSGA-II.

The population size is 40, the crossover probability is 0.9, the crossover distribution index is 10, and the mutation distribution index is 20. The evolutionary generation settings are delineated in Table 1. Figure 14 illustrates the simulation results from four experiments conducted using NSGA-II, where each point in the figure represents an individual solution. The coordinate settings are consistent with those employed for AMGA. As the number of iterations increases, a greater number of solutions converge on the Pareto front, exhibiting a clear convergence trend. The optimal solutions also exhibit a wider distribution range, better uniformity, and enhanced optimization performance.

**Figure 15:**
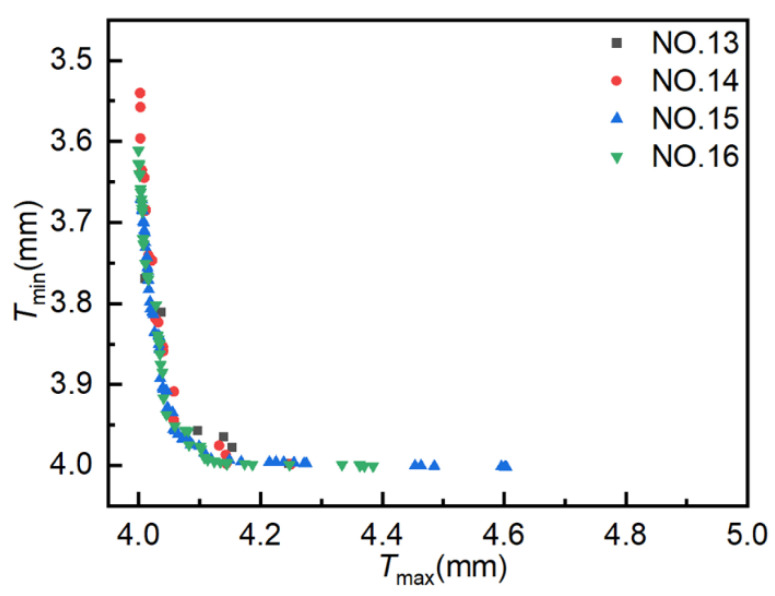
Pareto optimal solution under different experiments. Pareto optimal solutions obtained from NSGA-II experiments.

Figure 15 presents the distribution of Pareto optimal solutions across four experimental sets. The following observations can be seen from the figure: ① Results for 400, 800, 1200, and 1600 iterations yielded 6, 25, 64, and 64 Pareto optimal solutions, respectively. The number of Pareto optimal solutions increases with the number of iterations, but this increase is not significant in the later stages and may even decrease. ② As the number of iterations increases, the Pareto optimal solutions become increasingly close to the optimal solutions, with the Pareto optimal solutions obtained from Experiment NO. 16 being superior to those obtained from Experiment NO. 13. ③ A smaller number of iterations can also yield satisfactory Pareto optimal solutions, although the number of solutions is fewer, feasible results can be obtained quickly. ④ As the number of iterations increases, the distribution of optimal solutions becomes broader and more uniform. When the number of iterations increased from 1200 to 1600, the solutions in both cases converge along the Pareto front, and the number of optimal solutions was the same, so there was little room for further optimization. These results suggest that NSGA-II is effective for optimizing hydroforming process parameters, with 1200 iterations identified as the optimal trade-off between performance and computational cost.

### 4.5. Discussion

**Figure 16:**
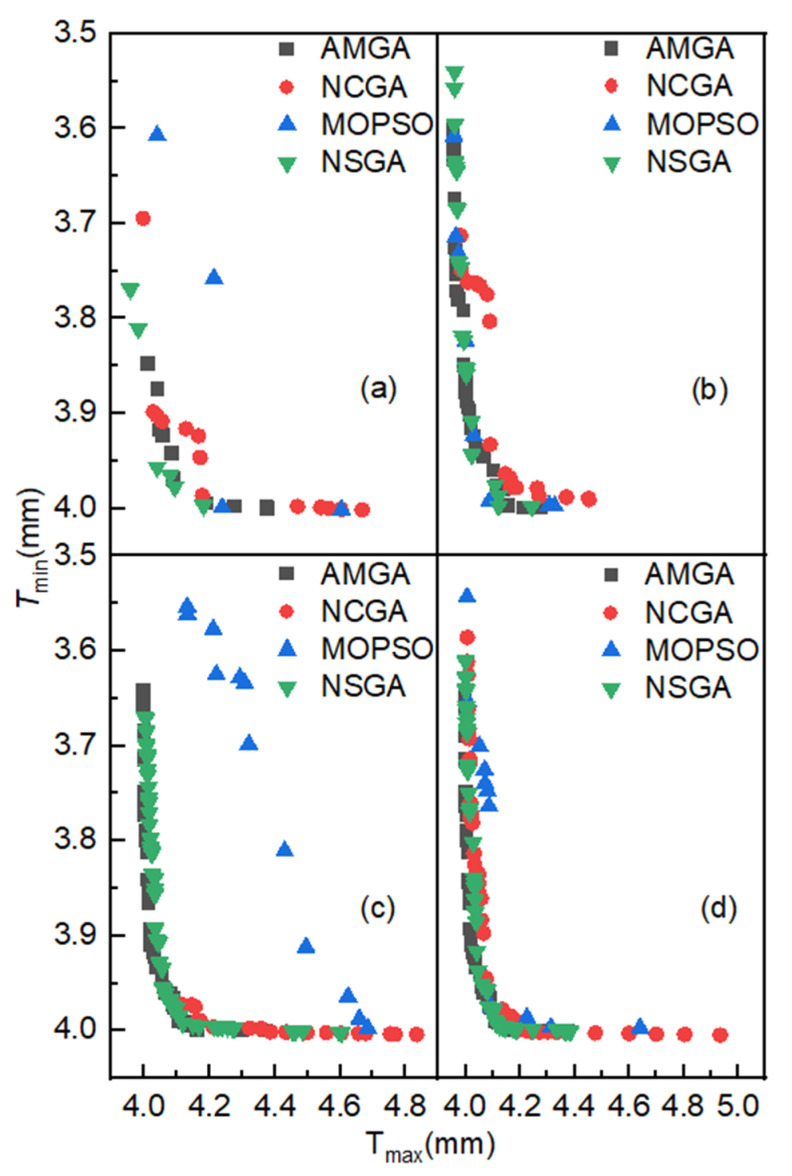
Comparison chart of Pareto optimal solution distribution under the same number of iterations: (**a**) 400 iterations; (**b**) 800 iterations; (**c**) 1200 iterations; (**d**) 1600 iterations.

Figure 16 compares algorithm performance through four diagram sets, each maintaining identical iteration counts but employing different algorithms. Critical findings include the following conclusions, which can be drawn from the analysis. Firstly, under the same number of iterations, the Pareto optimal solutions of NSGA-II and AMGA are more optimal. Secondly, as the number of iterations increases, the distribution range of the Pareto optimal solutions for AMGA, NCGA, and NSGA-II expands, with a greater number of solutions and better uniformity. Thirdly, to obtain a satisfactory set of Pareto optimal solutions, the number of iterations cannot be too small. It has been demonstrated that, upon surpassing a specified number of iterations, the solutions of AMGA, NCGA, and NSGA-II can be consolidated on the Pareto frontier, exhibiting a discernible convergence pattern. Furthermore, it has been observed that the application of NCGA for the optimization of hydroforming loading paths necessitates a greater number of iterations to achieve satisfactory outcomes. MOPSO demonstrates poorer suitability for hydroforming parameter optimization compared to NSGA-II, AMGA, and NCGA.

**Figure 17:**
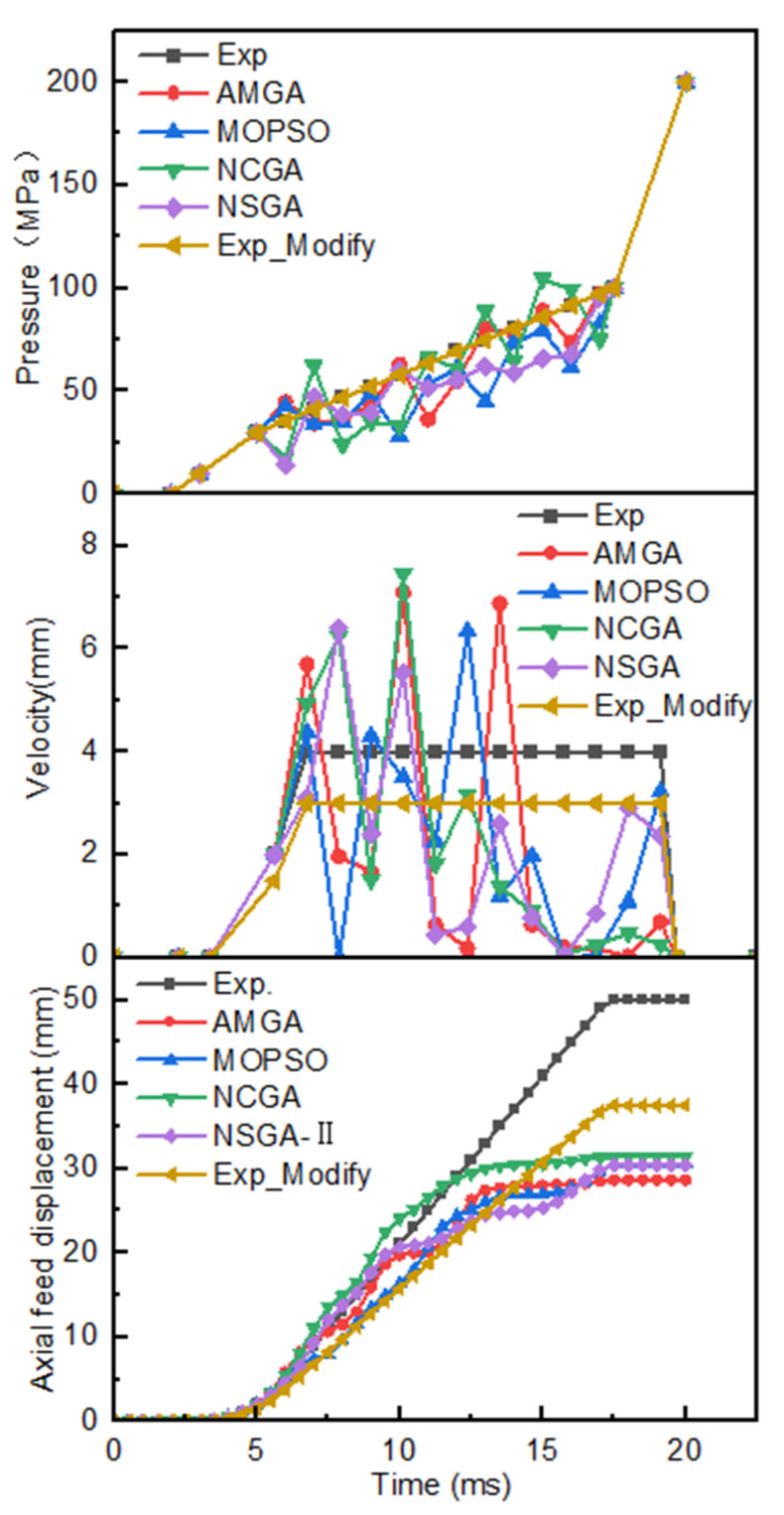
Pressure curve, feed velocity curve, and axial feed displacement under six schemes.

As illustrated in Figure 17, six hydroforming loading path curves are presented: the experience (Exp) curve, the AMGA-optimized curve, the NCGA-optimized curve, the MOPSO-optimized curve, the NSGA-II-optimized curve, and the Exp_Modify curve. While all six loading curves are nonlinear, the internal pressure and axial feed of the Exp curve and the Exp_Modify curve are linear during the forming stage. The four optimized internal pressure loading curves demonstrate fluctuations, oscillating above and below the Exp loading curve, with a reduced fluctuation range and an average value lower than that of the Exp curve. This finding suggests that the internal pressure in the Exp loading curve is comparatively high. The axial feed speeds of the four optimized curves also exhibit fluctuations, with a larger fluctuation range, and the axial feed rate in the middle and later stages is significantly lower than the Exp loading curve. Furthermore, the feed rates for the six optimized schemes are 50 mm, 28.7 mm, 30.5 mm, 31.4 mm, 30.4 mm, and 37.5 mm, respectively. The highest feed rate is observed for the Exp loading path, while the lowest is recorded for the AMGA optimization scheme. Following the modify of the Exp curve, the feed rate underwent a reduction from 50 mm to 37.5 mm, representing a 25% decrease. The feed rates of the four multi-objective optimization algorithms are comparable, with a maximum difference of less than or equal to 2.7 mm.

**Figure 18:**
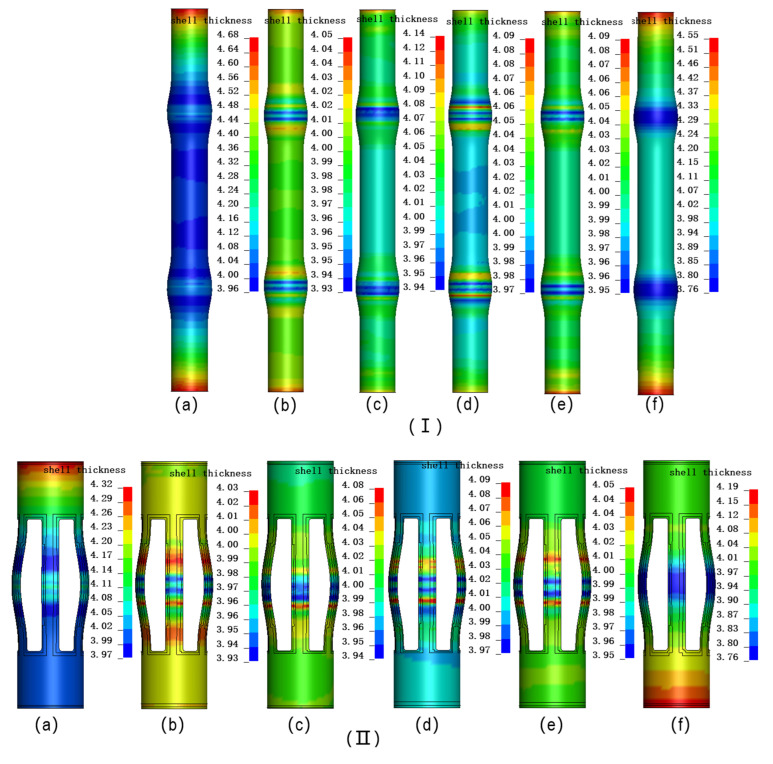
Simulation results using different process plans: (**a**) Exp.; (**b**) AMGA; (**c**) NCGA; (**d**) MOPSO; (**e**) NSGA-II; (**f**) Exp_Modify; (**I**) Simulation results after hydraulic expansion; (**II**) Simulation results after trimming and punching.

Figure 18I illustrates the wall thickness distribution cloud maps of the centralizer under six different schemes. The minimum wall thicknesses of the six schemes are 3.96 mm, 3.93 mm, 3.97 mm, 3.94 mm, 3.95 mm, and 3.76 mm, respectively, with corresponding thinning rates of 1%, 1.75%, 0.75%, 1.5%, and 4.63%, all within 5%; the maximum wall thicknesses of the tube blanks are 4.68 mm, 4.05 mm, 4.09 mm, 4.14 mm, 4.09 mm, and 4.55 mm, respectively, with corresponding wall thickness increase rates of 17%, 1.3%, 2.25%, 3.5%, 2.25%, and 13.75%, respectively. The following can be seen: ① The thickening rate of the Exp scheme is relatively high, but its thinning rate is very low, only 1%. ② Although the Exp_Modify scheme reduces the feed rate by 25%, the thickening rate remains at 13.7%, and the maximum thinning rate has increased significantly, from 1% to 4.5%. ③ The four multi-objective optimized loading paths perform better than the Exp loading path, and the obtained pipe blank wall thickness distribution is more uniform. ④ The thinning rates of the six schemes are all within 5%, and the pipe blanks can be successfully formed. Although the thickening rates of the Exp scheme and the Exp_Modify scheme both exceed 10%, the thickening parts are all at the ends, and the thickening rate will be reduced to a certain extent after trimming, which can ensure the mechanical performance of the centralizer.

Figure 18II presents the simulation results following trimming and punching. Compared to the parts before trimming and punching, the most significant changes were observed in the Exp_Modify scheme and the Exp scheme, where the maximum wall thickness of the tube blank decreased from 4.55 mm and 4.68 mm to 4.19 mm and 4.32 mm, respectively, with a reduction in wall thickness increase rate of 9%. However, the minimum wall thickness remained essentially unchanged. The maximum and minimum wall thickness changes for the other four schemes were negligible or nonexistent.

**Figure 19:**
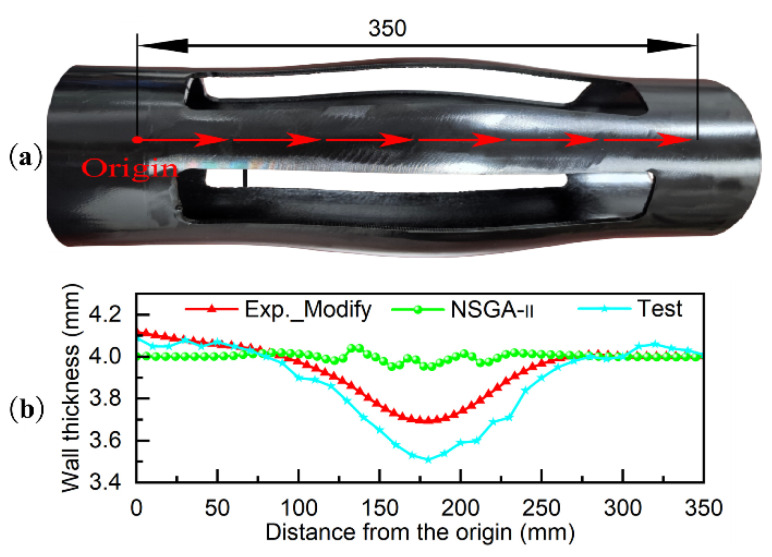
Comparison and analysis diagram of simulation results and test results: (**a**) Actual product view of the finished centralizer; (**b**) Thickness variation along the trajectory from simulation and test results.

Figure 19a,b present comparative analyses between the test locations on the sample parts, simulation results, and the measured test results of the samples, respectively. The following can be seen from the figures: (1) The thickness distribution trend measured by the sample is the same as the thickness distribution trend of the simulation analysis, with the maximum error occurring at the minimum wall thickness in the middle of the centralizer, which are 3.76 mm and 3.51 mm, respectively. The maximum thinning rates are 4.8% and 12.25%, respectively, with a difference of 7.37% between the two, which is less than the error of the pipe blank wall thickness. The simulation results are consistent with the actual measurement results, indicating that the simulation results are valid (note: when the seamless steel pipe wall thickness is 4 mm, the maximum error is ±10%). (2) Compared to the Exp scheme, the Exp_Modify scheme results in a more uniform wall thickness distribution of the tube blank. (3) Increasing the axial feed rate can effectively reduce the wall thickness reduction rate but causes a sharp increase in the thickening rate, resulting in a less uniform wall thickness distribution.

## 5. Conclusions

- The integration of NSGA-II, AMGA, and NCGA algorithms with LS-DYNA can achieve multi-objective optimization of hydroforming process parameters. With the same number of iterations, the Pareto optimal solutions of NSAG-II and AMGA are more optimal. In addition, NCGA requires more iterations to obtain a satisfactory Pareto optimal solution set.
- MOPSO demands a substantially larger number of iterations to optimize the hydroforming loading path; otherwise, it is easy to fall into a local optimum, and the convergence trend is not obvious.
- The number of iterations has a significant impact on the Pareto optimal solution. The more iterations, the closer the Pareto optimal solution is to the optimal solution, and the larger the solution set, the wider the distribution range, and the better the uniformity. An appropriate number of iterations can ensure that more Pareto optimal solutions are obtained, while also achieving better time efficiency and cost efficiency.
- The optimized method designed achieves automatic optimization through computers, greatly reducing the workload of designers and obtaining more optimal process parameters, providing a theoretical basis and basis for the selection of the optimization method for the loading path of the centralizer hydroforming.

## Figures and Tables

**Table 1:** Experimental design for optimization.

Serial Number	Algorithm	Population Size	Evolutionary Algebra	Number of Iterations
NO. 1	AMGA	40	-	400
NO. 2		40	-	800
NO. 3		40	-	1200
NO. 4		40	-	1600
NO. 5	NCGA	40	10	400
NO. 6		40	20	800
NO. 7		40	30	1200
NO. 8		40	40	1600
NO. 9	MOPS	10	40	400
NO. 10		10	80	800
NO. 11		10	120	1200
NO. 12		10	160	1600
NO. 13	NSGA-II	40	10	400
NO. 14		40	20	800
NO. 15		40	30	1200
NO. 16		40	40	1600

## Data Availability

The original contributions presented in this study are included in the article. Further inquiries can be directed to the corresponding authors.

## Abbreviations

The following abbreviations are used in this manuscript:

NSGA-II	Non-dominated Sorting Genetic Algorithm
MOPSO	Multi-Objective Particle Swarm Optimization
NCGA	Neighborhood Cultivation Genetic Algorithm
AMGA	Archive-based Micro Genetic Algorithm
ALE	Arbitrary Lagrange–Eulerian
DOE	Design of Experiment
ANN	Artificial Neural Networks
ε-MOABC	ε-dominance Multi-Objective Artificial Bee Colony
mm	Millimeters
Exp	Experience
